# Transient Developmental Purkinje Cell Axonal Torpedoes in Healthy and Ataxic Mouse Cerebellum

**DOI:** 10.3389/fncel.2016.00248

**Published:** 2016-11-02

**Authors:** Lovisa Ljungberg, Daneck Lang-Ouellette, Angela Yang, Sriram Jayabal, Sabrina Quilez, Alanna J. Watt

**Affiliations:** Department of Biology, McGill University, MontrealQC, Canada

**Keywords:** axon, pruning, microglia, synapse, myelin, neurofilament, ataxia, axonal swelling

## Abstract

Information is carried out of the cerebellar cortical microcircuit via action potentials propagated along Purkinje cell axons. In several human neurodegenerative diseases, focal axonal swellings on Purkinje cells – known as torpedoes – have been associated with Purkinje cell loss. Interestingly, torpedoes are also reported to appear transiently during development in rat cerebellum. The function of Purkinje cell axonal torpedoes in health as well as in disease is poorly understood. We investigated the properties of developmental torpedoes in the postnatal mouse cerebellum of wild-type and transgenic mice. We found that Purkinje cell axonal torpedoes transiently appeared on axons of Purkinje neurons, with the largest number of torpedoes observed at postnatal day 11 (P11). This was after peak developmental apoptosis had occurred, when Purkinje cell counts in a lobule were static, suggesting that most developmental torpedoes appear on axons of neurons that persist into adulthood. We found that developmental torpedoes were not associated with a presynaptic GABAergic marker, indicating that they are not synapses. They were seldom found at axonal collateral branch points, and lacked microglia enrichment, suggesting that they are unlikely to be involved in axonal refinement. Interestingly, we found several differences between developmental torpedoes and disease-related torpedoes: developmental torpedoes occurred largely on myelinated axons, and were not associated with changes in basket cell innervation on their parent soma. Disease-related torpedoes are typically reported to contain neurofilament; while the majority of developmental torpedoes did as well, a fraction of smaller developmental torpedoes did not. These differences indicate that developmental torpedoes may not be functionally identical to disease-related torpedoes. To study this further, we used a mouse model of spinocerebellar ataxia type 6 (SCA6), and found elevated disease-related torpedo number at 2 years. However, we found normal levels of developmental torpedoes in these mice. Our findings suggest that the transient emergence of Purkinje cell axonal torpedoes during the second postnatal week in mice represents a normal morphological feature in the developing cerebellar microcircuit.

## Introduction

Purkinje cell axons convey information away from the cerebellar cortical microcircuit, and are thus critical for cerebellar function. Over a century ago, focal axonal swellings were identified along Purkinje cell axons (Ramon and Cajal, 1991). The term “torpedo” was used to identify these focal swellings in 1918 by the Dutch psychiatrist Leendert Bouman (Bouman, 1918), and the term has been used since then to identify swellings or spheroids on Purkinje cell axons. Purkinje cell axonal torpedoes are observed in several diseases, including essential tremor (Louis et al., 2006, 2009, 2014), spinocerebellar ataxias (Sasaki et al., 1998; Yang et al., 2000; Louis et al., 2014), encephalopathy (Yagishita, 1978), and other cerebellar disorders (Hirano et al., 1973; Louis et al., 2014), and are especially prevalent in the cerebellar vermis (Louis et al., 2011). Torpedo-like swellings have also been observed in several spontaneously arising ataxic rodents, for e.g., *weaver* (Hirano et al., 1973), hyperspiny Purkinje cell (hpc) (Sotelo, 1990), and *sticky* mice (Sarna and Hawkes, 2011), *groggy* rats (Takeuchi et al., 1995), and in mouse models of disease such as Autosomal Recessive Ataxia of the Charlevoix-Saguenay Region (ARSACS) (Lariviere et al., 2015). Furthermore, torpedoes are enriched in rodent brains after chronic administration of certain chemicals, such as the anti-seizure medicine phenytoin (Volk and Kirchgassner, 1985), the excitotoxic kainic acid (Rossi et al., 1994), and molecules that interfere with microtubule transport (Pioro and Cuello, 1988). Purkinje cell axonal torpedoes have also been observed close to cerebellar lesions (Takahashi et al., 1992). Taken together, these observations have led to the belief that Purkinje cell axonal torpedoes are associated with cerebellar damage and degeneration. Indeed, torpedoes can be observed on the axons of surviving cells at the same time as Purkinje cell death is observed (Louis et al., 2014). This suggests that the relationship between cell death and Purkinje cell torpedo accumulation is complex. For example, torpedoes are numerous in the cerebella from essential tremor patients who have significant Purkinje cell loss, suggesting that torpedoes are prevalent on axons of Purkinje cell that do not die. However, in diseases such as multiple system atrophy-cerebellar, torpedoes are more prevalent when Purkinje cell loss is minimal. Multiple system atrophy-cerebellar patients that have greater Purkinje cell loss have fewer torpedoes, possibly because the neurons with torpedoes have died (Louis et al., 2014). It is thus an open question whether torpedoes cause neurodegeneration or are in fact neuroprotective (Babij et al., 2013). Interestingly, torpedoes also occur in healthy brains (Kato and Hirano, 1985), and there is some evidence that torpedoes accumulate with age in both human and rodent cerebellum (Baurle and Grusser-Cornehls, 1994). The presence of torpedoes in aging cerebellum may occur because of the accumulation of changes that are similar to those observed in neurodegenerative diseases but in an age-dependent manner.

In addition to torpedoes being prevalent in diseased and aged brains, focal swellings on Purkinje cell axons that at least superficially resemble Purkinje cell torpedoes have been observed in the developing rat, with a transient peak observed from the second to third postnatal week of development (Gravel et al., 1986). Even less is known about the properties or functions of these so-called developmental torpedoes.

We use a transgenic mouse that expresses an enhanced GFP fused to tau (Sekirnjak et al., 2003), which brightly labels Purkinje cell axons (Watt et al., 2009), to characterize developmental Purkinje cell torpedoes in mice. We find that developmental torpedoes are observed in the second and third postnatal week of development, at ages after developmental Purkinje cell death has occurred, and the total number of Purkinje cells is static. Purkinje cell developmental torpedoes are seldom associated with a collateral branch point, and microglia are not enriched around developmental torpedoes, suggesting that they are not likely to be associated with axonal pruning. They do not stain for an inhibitory presynaptic marker, which suggests that they are not the presynaptic element of transient inhibitory synapses. Developmental torpedoes appear largely on myelinated axons and, like disease-related torpedoes, most are enriched with neurofilament, although a sizeable fraction of neurofilament-negative developmental torpedoes were also observed. Finally, we report that although Purkinje cells had elevated number of torpedoes in a mouse model of spinocerebellar ataxia type 6 in aged mice, we found no differences in the number of developmental torpedoes in these mice, or when motor deficits are first observed. Taken together, these results suggest that developmental torpedoes are not pathophysiological but rather represent a normal morphological Purkinje cell axonal feature during the formation of the cerebellar microcircuit.

## Materials and Methods

### Animals and Slice Preparation

We used L7-tau-gfp mice (Sekirnjak et al., 2003; Watt et al., 2009) (generous gifts from both S. du Lac and M. Häusser), and C57BL6/J mice (purchased from Jackson laboratories, Bar Harbor, ME, USA) to characterize developmental torpedoes. For the SCA6^84Q/84Q^ disease model we used transgenic mice harboring a humanized 84-CAG repeat tract in the mouse *Cacna1a* locus (Jackson laboratories; strain B6.129S7-Cacna1a^tm3Hzo^/J; stock number: 008683) (Watase et al., 2008). Mice [postnatal day (P)5-P15] were anesthetized with isoflurane, sacrificed, and the brain was rapidly removed into ice-cold ACSF (in mM) 125 NaCl, 2.5 KCl, 2 CaCl2, 1 MgCl2, 1.25 NaH2PO4, 26 NaHCO3, and 20 glucose. Mice (P15 and older) were transcardially perfused with 4% PFA (EMS, Hatfield, PA, USA) and the brains were quickly removed. In some cases, P30 mice were anesthetized with isoflurane, sacrificed, and the brain was rapidly removed into an ice-cold partial sucrose replacement solution (in mM) 111 Sucrose, 50 NaCl, 2.5 KCl, 0.65 CaCl2, 10 MgCl2, 1.25 NaH2PO4, 25 NaHCO3, and 25 glucose. For mice of all ages, the brains extracted (as above) were then quickly transferred into 4% PFA (EMS, Hartfield, PA, USA), and stored at 4°C on a rotary shaker at 70 RPM for at least 48 h, then transferred to PBS with 0.05% sodium azide. Results were compared for tissue prepared with and without perfusion, but no significant changes were observed and this data was pooled.

The vermis of the cerebellum was then submerged in 0.05% sodium azide in 0.01 M PBS and sliced into 100 μm parasagittal slices on a Leica Vibratome 3000 plus (Concord, ON, Canada). The slices were stored at 4°C in 0.05% sodium azide in 0.01 M PBS. Animal procedures were approved by the McGill Animal Care Committee and conform to the guidelines set in place by the Canadian Council on Animal Care.

### Immunocytochemistry

All staining was performed in a blocking solution with 5% BSA, 0.05% sodium azide, and 0.4% Triton X in 0.01 M PBS. The primary antibodies used were mouse anti-Neurofilament 200 kD (1:500, Millipore, Temecula, CA, USA; Cat. No.: MAB5266), mouse anti-Myelin Basic Protein (MBP; 1:500, Biolegend, San Diego, CA, USA; Cat. No.: 836501), rabbit anti-Green Fluorescent Protein (1:500, Millipore, Temecula, CA, USA; Cat. No.: AB3080P) (in P5 and P7 animals to amplify the GFP signal), guinea pig anti- (1:500, Cedarlane, Burlington, Ontario, Canada; Cat. No.: 131004(SY)) rabbit anti-Iba1 (1:500, Wako Chemicals, Osaka, Japan, Cat. No.: 019-19741), and rabbit anti-Calbindin D-28k (1:1000, Swant, Switzerland; Cat. No.: CB38). The slices were incubated in primary antibody at 4°C for 72 h on a rotary shaker at 70 RPM. Slices were then rinsed three times in a solution containing 0.4% Triton X in 0.01 M PBS.

The secondary antibodies were used at dilutions corresponding to the primary antibody. The secondary antibodies used were Alexa Fluor 594 donkey anti-rabbit (Jackson ImmunoResearch, West Grove, PA, USA; Cat. No.: 711-585-152), Alexa Fluor 488 donkey anti-mouse (Jackson ImmunoResearch; Cat. No.: 715-545-150), Alexa Fluor 594 donkey anti-guinea pig (Jackson ImmunoResearch; Cat. No.: 706-585-1480) and Alexa Fluor 594 donkey anti-mouse (Jackson ImmunoResearch; Cat. No.: 715-585-10). AffiniPure Fab Fragment donkey anti-mouse (Jackson ImmunoResearch; Cat. No.: 715-007-003) was used during secondary staining with antibodies made in mouse to avoid cross reactivity. The secondary staining was performed at 4°C for 90 min on a rotary shaker at 70 RPM. Slices were rinsed with 0.4% Triton X in 0.01 M PBS, mounted onto slides with Prolong Gold Antifade mounting media (Invitrogen), and stored in the dark at 4°C. Chemicals were purchased from Sigma unless otherwise indicated.

### Imaging

**FIGURE 1:**
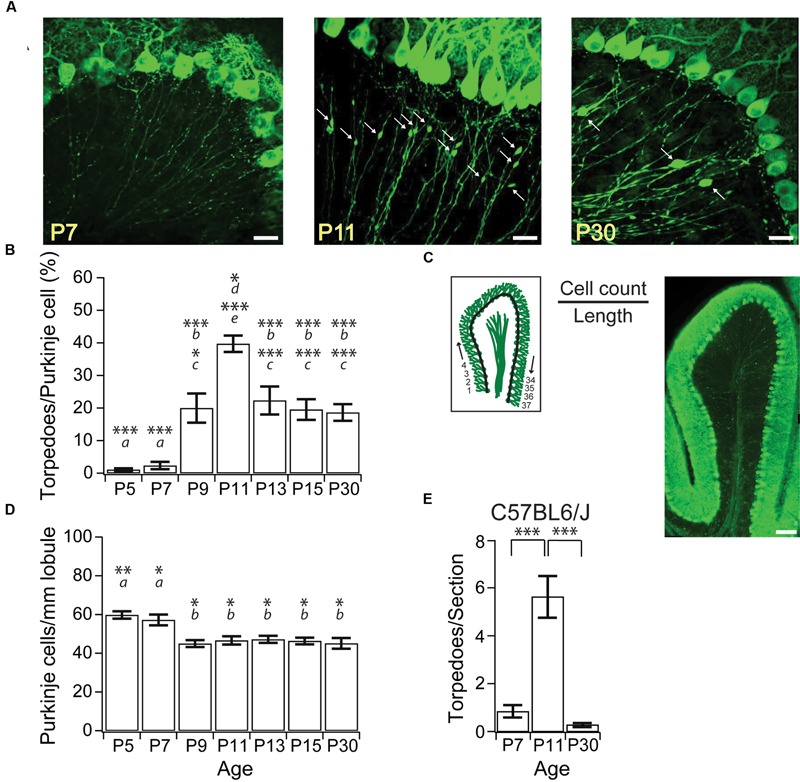
**Purkinje cell axonal torpedoes peak at P11 during postnatal mouse development. (A)** Sample images highlighting Purkinje cell axons at P7 (left), P11 (middle), and P30 (right) from L7-tau-GFP mice. White arrows point to torpedoes. Note the abundance of Purkinje cell axon torpedoes at P11. Scale bar, 20 μm. **(B)** Summary data showing number of torpedoes peak at P11, with nearly 40% of Purkinje cell soma with a torpedo. Torpedoes are nearly absent during first week (P5-7) of development. Even at P30, there are a significant number of Purkinje cells that harbor axon torpedoes. **(C)** Left, illustration of how cell density measurements were taken. The number of Purkinje cells per length of lobule were counted for the entire Lobule III to calculate total Purkinje cell density, cell count/length. Right, sample image of sagittal slice through lobule III. Note that there are torpedoes present in the white matter that have not been included in our analysis. Scale bar, 100 μm. **(D)** Purkinje cell density (cells/mm lobule) in Lobule III is higher at P5 and P7, but remains constant after P9, consistent with reports that apoptosis is over by P9 in developing mouse Purkinje cells (Jankowski et al., 2009). **(E)** Torpedoes were also transiently enriched in P11 C57BL6/J wild-type (WT) cerebellum, and were low at both P7 and P30. Significance determined by Wilcoxon multiple comparisons followed by Benjamini–Hochberg procedure with the FDR of 0.05 for panel **(B,E)**; significance determined by one-way ANOVA followed by Tukey HSD test for panel **(D)**. Asterisks denote the minimum significance for comparisons where the letter denotes the relevant comparisons: ***a*** = significantly different from P9, P11, P13, P15, and P30; ***b*** = significantly different from P5 and P7; ***c*** = significantly different from P11; ***d*** = significantly different from P9; ***e*** = significantly different from P5, P7, P13, P15, and P30. All comparisons that are non-indicated are not significantly different, *P* > 0.05. ^∗^*P* < 0.05; ^∗∗^*P* < 0.01; ^∗∗∗^*P* < 0.005.

Slices were imaged with a custom-built two-photon microscope with a Ti:Sapphire laser (MaiTai; Spectra Physics, Santa Clara, CA, USA) tuned to either 890 nm (GFP) or 775 nm (non-GFP). Image acquisition was done using ScanImage running in Matlab (Mathworks, Natick, MA, USA) (Pologruto et al., 2003). All imaging was done in lobule III of the cerebellar vermis. Whole lobule images (**Figure 1**) were obtained with a Leica upright fluorescent microscope using GFP filter.

### Data Analysis

Image analysis was performed in ImageJ. Torpedo density was measured as number of torpedoes/number of Purkinje cells per acquisition when signal to noise was high. However, for very young slices and very old slices, we found that Calbindin labeling gave high background, and we thus counted the number of torpedoes/section instead. Torpedo length was measured as the distance from the beginning to the end of the swelling parallel to the axon. Torpedo width was measured perpendicular to the length, and close to the widest point of the swelling. Axial ratio was defined as the length/width of each torpedo. To compare the density of microglia around torpedoes and axons, a 25 μm × 25 μm box was centered around either Purkinje cell axonal torpedoes or Purkinje cell axons without neighboring torpedoes while viewing only the green (Purkinje cell label) channel. Switching to the red channel, the red-labeled microglia that were in the boxed region were counted. To strengthen our analytical power, we performed analysis in tandem for key findings, including developmental torpedo count. Two individuals analyzed data images independently, and results were found to be consistent across experimenter. Data was collected from equal number of acquisitions from at least three animals from a minimum of two litters (typically three) for each comparison.

### Statistics

Data are reported as Mean ± SEM or as percentage of totals for distributions. Comparisons were made using one-tailed ANOVA tests followed by Tukey’s HSD test for normal distributions in JMP software (SAS, Cary, NC, USA), or Student’s *t*-tests in Igor Pro (Wavemetrics, Portland, OR, USA). For non-normal distributions, we used Wilcoxon/Kruskal–Wallis multiple comparison tests followed by the Benjamini–Hochberg procedure using a false discovery rate (FDR) of 0.05 in JMP software (SAS, Cary, NC, USA) or a Mann–Whitney *U* test in Igor Pro. Multiple-comparison corrections were performed with Excel scripts. *P*-values of <0.05 were considered significant.

## Results

### Developmentally Transient Torpedoes on Axons of Purkinje Cells that Persist into Adulthood

Focal axonal swellings, or torpedoes, have been observed on the axons of developing Purkinje cell from postnatal rats (Gravel et al., 1986), and have been reported in developing mice (Baurle and Grusser-Cornehls, 1994). To characterize developmental axonal torpedoes found in the developing mouse cerebellum on Purkinje cell axons, we imaged Purkinje cell axons from several postnatal ages (**Figure 1A**) from L7-tau-GFP mice that express a tau-GFP fusion protein in Purkinje cells, resulting in strong axonal labeling, as previously described (Sekirnjak et al., 2003; Watt et al., 2009). To determine how prevalent developmental torpedoes were, we counted their numbers normalized to Purkinje cell number at several ages from both the apex and bank of anterior Lobule III of the vermis. We observed a rapid and transient increase in large focal axonal swellings, or developmental torpedoes, which were largely absent before P9, peaked at P11, and had decreased significantly by P30 (**Figures 1A,B**; P5: 7 torpedoes counted/702 Purkinje cells total, or 1.1 ± 0.44% normalized to acquisition, see “Materials and Methods” for details; P7: 16 torpedoes/690 Purkinje cells or 2.4 ± 1.1%; P9: 140 torpedoes/736 Purkinje cells, or 20.0 ± 4.5%; P11: 273 torpedoes/674 Purkinje cells, or 39.7 ± 2.5%; P13: 140 torpedoes/633 Purkinje cells, or 22.4 ± 4.3%; P15: 145 torpedoes/766 Purkinje cells or 19.5 ± 3.2%; P30: 141 torpedoes/774 Purkinje cells, or 18.6 ± 2.6%). Developmental torpedoes could be observed along Purkinje cell axons in both the granule cell layer and white matter (**Figures 1A,C**); however, because of the bundling of the Purkinje cell axons in white matter, which makes individual axons and torpedoes difficult to resolve, we limited our analysis to Purkinje cell axons in the granule cell layer. Interestingly, although torpedoes were not observed in younger ages, Purkinje cell axons in the first postnatal week displayed multiple small varicosities, as previously reported (Baurle and Grusser-Cornehls, 1994), which were distinguishable from torpedoes based on their smaller size (compare **Figure 1A**, left, middle). We thus restricted our analysis to developmental torpedoes, which showed a similar time course to those reported in rat (Gravel et al., 1986).

We wondered whether the transient changes in Purkinje cell torpedoes we observed were due to the presence of the tau-GFP protein in our transgenic mice, since tau is important for axonal integrity, and overexpressing tau can be pathological in Alzheimer Disease (Duan et al., 2012; Krstic and Knuesel, 2013). To determine whether our results were influenced by overexpression of tau, we examined Purkinje cell axons from C57BL6/J mice and counted the number of developmental torpedoes at three different ages: P7, P11, and P30 (**Figure 1E**; P7: 0.84 ± 0.26 torpedoes/section; P11: 5.6 ± 1.2 torpedoes/section; P30: 0.29 ± 0.08 torpedoes/section). Broadly speaking, we observed a similar shape in the developmental profile of torpedoes from C57BL6/J mice as in L7-tau-GFP mice, with a peak of torpedoes at P11 (significantly different from P7 and P30; *P* < 0.0001). These values are lower than corresponding torpedo counts from L7-tau-GFP mice (P7: 1.1 ± 0.42 torpedoes/section; P11: 18.4 ± 1.7 torpedoes/section; P30: 8.8 ± 1.2 torpedoes/section, data not shown). However, we cannot directly compare these values since image quality varies for calbindin antibody labeling and endogenous GFP expression, and it is possible that calbindin does not label all torpedoes. To address this, we processed a subset of L7-tau-GFP sections for calbindin and counted the number of torpedoes that we observed. Remarkably, we detected only 46.4% of torpedoes with calbindin compared to the same sections examined in the GFP channel (*N* = 15 sections). This suggests that either only a subset of torpedoes are labeled with calbindin, and/or we detect fewer torpedoes with immunocytochemistry due to poorer image quality. Thus, our data indicates that L7-tau-GFP is a good model to study developmental torpedoes because more torpedoes can be detected than with standard immunocytochemistry. Although it is possible that the presence of tau increases torpedo numbers in developing axons, this likely accounts for a relatively small proportion of torpedoes at these ages.

Purkinje cell axon torpedoes have been observed in diseased (Louis et al., 2006, 2009, 2014) and aged cerebellum (Baurle and Grusser-Cornehls, 1994), where they have often been associated with Purkinje cell death (Louis et al., 2014). We wondered whether developmental torpedoes might also be related to Purkinje cell death, since developmental apoptosis of Purkinje cells is a normal part of cerebellar development (Light et al., 2002; Jankowski et al., 2009). To address this question, we measured the number of Purkinje cells in a sagittal slice from an entire lobule III at several ages (**Figure 1C**), and found that Purkinje cell numbers were elevated at both P5 and P7, but remained constant after P9 (P5: 59.8 ± 1.9 Purkinje cells/mm; P7: 57.2 ± 2.8 Purkinje cells/mm; P9: 45.0 ± 1.8 × 10^-2^ Purkinje cells/mm; P11: 46.7 ± 2.1 Purkinje cells/mm; P13: 47.2 ± 1.8 Purkinje cells/mm; P15: 46.4 ± 1.7 Purkinje cells/mm; P30: 45.1 ± 2.7 Purkinje cells/mm; at least four whole-lobule images from four mice included at each age point). Our findings are in agreement with previous reports demonstrating that developmental Purkinje cell apoptosis peaks at P3 and is complete by P9 (Light et al., 2002; Jankowski et al., 2009). Our results argue that the majority of developmental torpedoes occur on axons of Purkinje cells that will persist into adulthood, since they appear after the peak of Purkinje cell apoptosis. However, the small fraction of torpedoes that appear at or before P9 might arise on axons of Purkinje cells that will undergo developmental apoptosis.

### Developmental Torpedoes Are Unlikely to be Associated with Axonal Pruning

**FIGURE 2:**
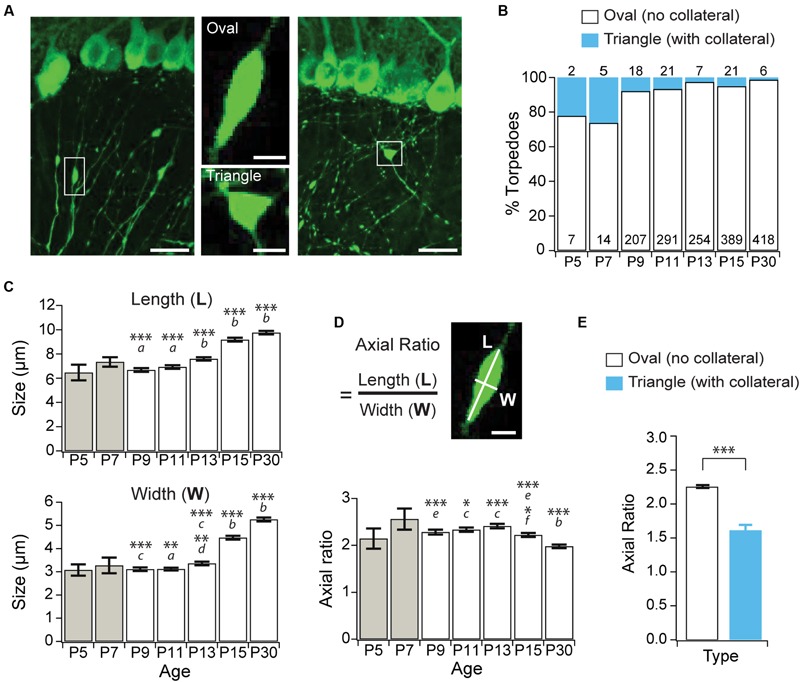
**Purkinje cell developmental torpedoes are seldom associated with axonal branch points. (A)** Left: sample image showing oval-shaped axonal torpedo (inset, middle top); and right: triangle-shaped torpedo associated with axonal collateral branch point (inset, middle bottom). Scale bar for left and right images, 20 μm; scale bar for insets in middle, 5 μm. **(B)** The majority of torpedoes are not associated with collateral branch points (white bars), with only a subset associated with collaterals (blue bars) at all ages. Number of torpedoes counted indicated in (white) or above (cyan) bars. **(C)** Torpedo length (top) and width (bottom) increased after P13. **(D)** The axial ratio (length, *L*/width, *W*) significantly decreased at P15 and P30 because torpedo width increased more than length at these ages. Scale bar, 5 μm. **(E)** The axial ratio is significantly lower in triangle torpedoes associated with axon collateral branch points than in oval-shaped torpedoes that are not associated with branch points. Note that because there are so few torpedoes at P5 and P7 **(B)**, these ages were not included in the analysis in panels **(C,D)**. They are included in the graph for reference (gray bars). Significance determined by Wilcoxon multiple comparisons followed by Benjamini–Hochberg procedure with the FDR = 0.05 for panels **(C,D)**, and with a Mann–Whitney *U* test for panel **(E)**. Asterisks denote the minimum significance for comparisons where the letter denotes the relevant comparisons: ***a*** = significantly different from P13, P15, and P30; ***b*** = significantly different from all other ages; ***c*** = significantly different from P15, and P30; ***d*** = significantly different from P11; ***e*** = significantly different from P30. ***f*** = significantly different from P11 and P13. All comparisons that are non-indicated are not significantly different, *P* > 0.05. ^∗^*P* < 0.05, ^∗∗^*P* < 0.01; ^∗∗∗^*P* < 0.001.

Purkinje cell axons extend collaterals that are extensively pruned during the second week of postnatal development (Gianola et al., 2003), and we wondered whether developmental torpedoes are associated with axonal collateral branch points. Collateral-associated torpedoes are structurally distinct, with a triangular rather than oval shape (**Figure 2A**). To address this, we counted how often Purkinje cell torpedoes were associated with an axon collateral (**Figures 2A,B**), and found that only a small fraction of torpedoes are located at collateral branch points (**Figures 2A,B**). Disease-related torpedoes have been characterized morphologically using the axial ratio measurement: length/width of torpedoes (Baurle and Grusser-Cornehls, 1994). We measured the length and width of torpedoes throughout postnatal mouse development (**Figure 2C**), and calculated their axial ratio (**Figure 2D**). We found that both the length and width of torpedoes increased with age (**Figure 2C**) while the axial ratio decreases from P15 onward (**Figure 2D**), suggesting that the torpedoes that persist after P11 increase in width more rapidly than they increase in length. Consistent with their different shape, the axial ratio of the collateral-branch-point-associated triangular torpedoes is significantly lower than non-collateral associated oval torpedoes (**Figure 2E**). Thus, developmental axonal torpedoes are typically not associated with axonal collaterals, and are structurally dynamic across development.

**FIGURE 3:**
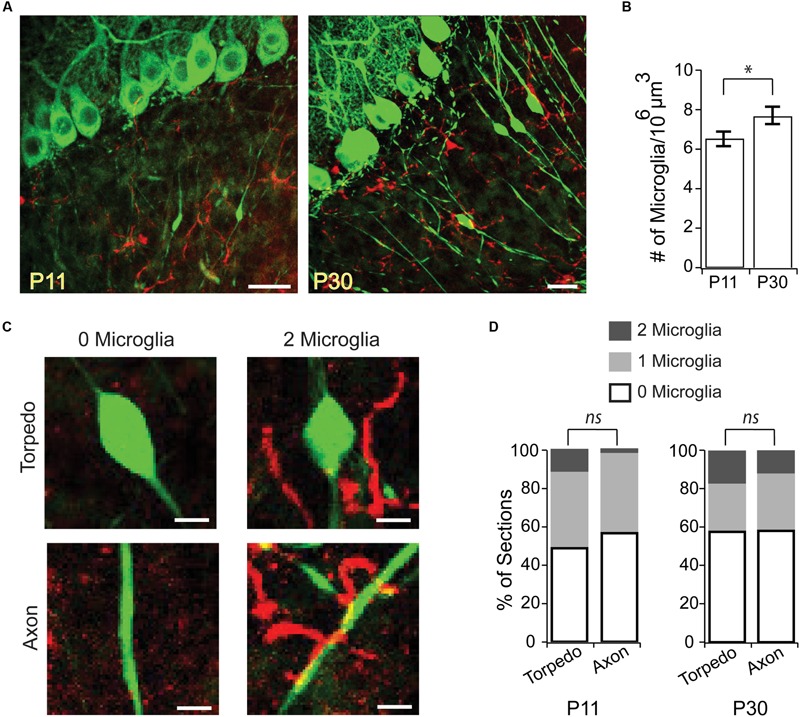
**Microglia are not enriched around developmental torpedoes. (A)** Sample images from P11 (left) and P30 (right) of Purkinje cells and their axons (green, eGFP), and activated microglia (red, Alexa 594). Scale bar, 20 μm. **(B)** The density of activated microglia increases in the developing granule cell layer between P11 and P30. **(C)** Sample images highlighting how analysis was done. After identifying stretches of axon with torpedoes (top) or without torpedoes (bottom) in the green channel, the red channel was added and the number of nearby microglia (within a 25 μm^3^ box centered on the torpedo or axon) were counted: merged examples show 0 microglia (left) and 2 microglia (right). Scale bar, 5 μm. **(D)** Number of microglia in the vicinity of torpedo or axon, from 0 to 2 microglia/section (white–gray). No significant differences were observed between microglia density around torpedoes or non-torpedo axons at either P11 (left; *P* = 0.84), or P30 (right; *P* = 0.84). Significance determined by Student’s *t*-test **(B)** or Mann–Whitney *U* test **(D)**. ^∗^*P* < 0.05, *ns P* > 0.05.

Could Purkinje cell torpedoes be associated with sites of axonal remodeling without being directly associated with a branch point? For example, could the swelling of a torpedo mark where a recent axonal collateral has been pruned? To address this, we examined the localization of activated microglia in the granule cell layer, which have been associated with axonal refinement and pruning during development (Pont-Lezica et al., 2011), and can be recruited to axonal swellings (di Penta et al., 2013; Kato et al., 2016). We looked at two ages: at P11, the peak of developmental torpedo density, and at P30 (**Figure 3A**), when torpedoes are larger but less numerous. We found that microglia density in the granule cell layer increased significantly over this period (**Figure 3B**; P11: 6.52 ± 0.37 cells/10^6^ μm^3^; P30: 7.71 ± 0.44 cells/10^6^ μm^3^; significantly different, *P* = 0.045), likely because microglia density throughout the cerebellum overall increases across development (Perez-Pouchoulen et al., 2015). To determine whether microglia were enriched around torpedoes, which might suggest that they are involved in axonal refinement, we measured the local density of microglia around torpedoes (**Figure 3C**) and compared this density to that around Purkinje cell axons that are not in the proximity of a torpedo (**Figure 3C** shows two examples for each). We found no significant enrichment of microglia around torpedoes compared to axons at either the peak age of torpedo enrichment, P11 (**Figure 3D**, left; P11 torpedo: 25/51 or 49.0% have 0 microglia in proximity, 20/51 or 39.2% are near 1 microglia, and 6/51 or 11.8% are near 2 microglia; P11 axon: 29/51, or 56.9% have 0 microglia in proximity, 21/51 or 41.2% are near 1 microglia, and 1/51 or 1.9% are near 2 microglia; not significantly different *P* = 0.84) or later when torpedoes are less prevalent but larger, P30 (**Figure 3D**, right; P30 torpedo: 11/45 or 24.4% have 0 microglia in proximity, 26/45 or 57.8% are near 1 microglia, and 8/45 or 17.8% are near 2 microglia; P30 axon: 14/48, or 56.9% have 0 microglia in proximity, 28/48 or 58.3% are near 1 microglia, and 6/48 or 12.5% are near 2 microglia; not significantly different *P* = 0.84). Taken together, our findings suggest that torpedoes are unlikely to be associated with axonal damage or refinement.

### Developmental Torpedoes Are Unlikely to be Presynaptic Terminals

**FIGURE 4:**
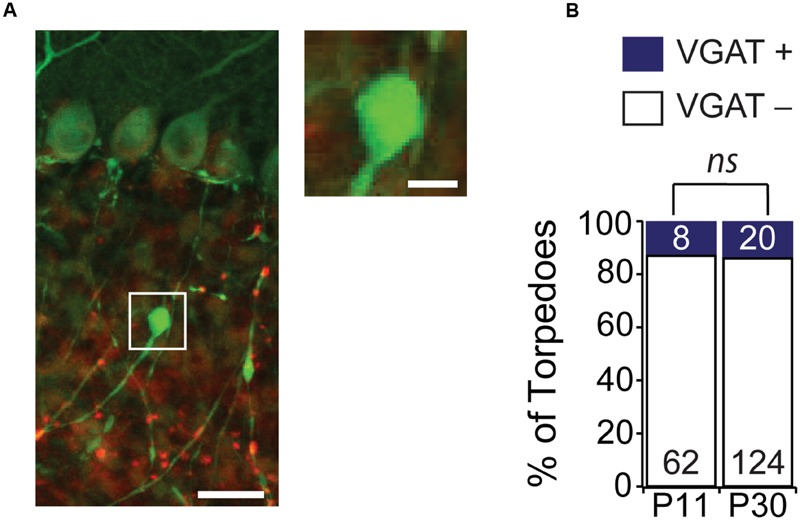
**Purkinje cell torpedoes unlikely to be presynaptic terminals. (A)** Sample image of Purkinje cell with torpedo (green) that is co-stained with the presynaptic inhibitory terminal marker VGAT (red). Inset shows that torpedo does not label for VGAT. Scale bar for left image, 20 μm; scale bar for insets to right, 5 μm. **(B)** The vast majority of torpedoes at both P11 and P30 are not positive for VGAT (white bars) with only a subset having any VGAT labeling nearby (VGAT+, blue bars), suggesting that they are unlikely to be presynaptic terminals. Number of torpedoes (N) counted is indicated in bars. Significance determined by Mann–Whitney *U* test, *ns P* > 0.05.

Some neurons, such as hippocampal dentate gyrus granule cells, have striking focal axonal swellings along their axons that are the morphological correlates of large presynaptic terminals (Chamberland et al., 2014). These large presynaptic terminals bear a resemblance to Purkinje cell axonal torpedoes. Although ultrastructure analysis of disease-related Purkinje cell axonal torpedoes reveals that they do not contain vesicles (Petito et al., 1973; Yagishita, 1978; Mann et al., 1980; Louis et al., 2009), suggesting that they are not presynaptic release sites, we wondered whether developmental Purkinje cell axonal torpedoes might be the presynaptic structure of a transient synapse that functions during postnatal development. Interestingly, Purkinje cells form transient synapses onto other Purkinje cells during postnatal development (Watt et al., 2009), and transient synapses are thought to be a common feature of developing brain circuits (van Welie et al., 2011), and can also be found in other cerebellar neurons (Trigo et al., 2010). To examine whether developmental torpedoes were axon terminals containing vesicles, we used immunolabeling for vesicular GABA transporter (VGAT; **Figure 4A**) (Watt et al., 2009), and quantified the number of torpedoes that colocalized with this presynaptic marker (**Figures 4A,B**). We found that the vast majority (>80%) of Purkinje cell torpedoes were negative for VGAT staining, both at P11 and P30 (**Figure 4B**; P11: 54/62, or 87.1% of torpedoes were VGAT negative; P30: 124/144, or 86.1% of torpedoes were VGAT negative, not significantly different, *P* = 0.62), which suggests that like disease-related torpedoes, developmental torpedoes do not appear to be inhibitory presynaptic terminals.

### Structural Distinctions between Developmental and Disease-Related Torpedoes

**FIGURE 5:**
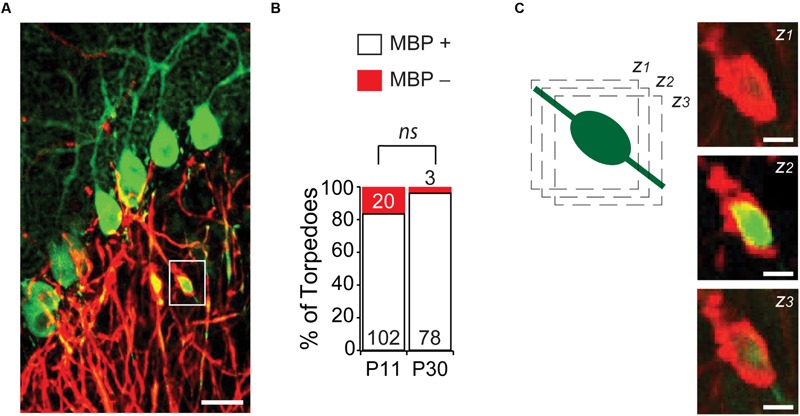
**Most developmental torpedoes are myelinated. (A)** Sample image showing Purkinje cells and axons (green) as well as myelin basic protein (MBP; red). Scale bar, 20 μm. **(B)** The vast majority of torpedoes at both P11 and P30 are positive for MBP (white bars), with only a fraction negative for MBP (red). Number of torpedoes (N) is indicated in or above each bar. **(C)** Successive z-stack images (z*_1-3_*) of region highlighted in panel **(A)**, highlighting that myelin appears to fully wrap around Purkinje axon developmental torpedo. Scale bar, 5 μm. Significance determined by Mann–Whitney *U* test, *ns P* > 0.05.

An understanding of Purkinje cell developmental torpedo function is currently unknown. Since the myelination of Purkinje cell axons is underway during the second postnatal week of development (Gianola et al., 2003), at the age when developmental torpedoes are prevalent, we wondered whether developmental torpedoes occur on myelinated or unmyelinated axons. To examine this, we used MBP to identify myelin, and examined whether torpedoes were surrounded by myelin (MBP+) or not (MBP-; **Figures 5A,B**), at P11, the peak of torpedo density, and at P30, when torpedo numbers are decreased and when Purkinje cell axons are expected to be fully myelinated (Gianola et al., 2003). We found that the vast majority of torpedoes at P11 (102/122, or 85%) and at P30 (78/81, or 96%) were myelinated (**Figure 5B**), with no significant difference in the percentage of non-myelinated axons across ages (*P* = 0.13). While we considered any local enrichment of MBP to be positive for myelin, we typically observed torpedoes to have good myelin coverage (**Figure 5C** shows three successive z-stack images). This suggests that action potential conductance may not be hindered in developing axons by the presence of a developmental axonal torpedo, although it is possible that we missed subtle changes in myelin that could affect propagation. We found no significant differences in the length (*P* = 0.41), width (*P* = 0.60), or axial ratio (*P* = 0.62) of myelinated or unmyelinated torpedoes at P11 (there were too few non-myelinated torpedoes to compare at P30), suggesting that these torpedoes are not distinct subpopulations. Rather, whether a developmental torpedo is myelinated or not is likely to depend on whether its parent axon is myelinated or not, and not on the torpedo. The high percentage of developmental torpedoes that are myelinated is in contrast to what is observed for disease-related torpedoes, which although occurring on both myelinated or non-myelinated axons, (Yagishita, 1978; Takeuchi et al., 1995; Louis et al., 2009), disease-related torpedoes are typically more common on non-myelinated axons (Louis et al., 2009).

**FIGURE 6:**
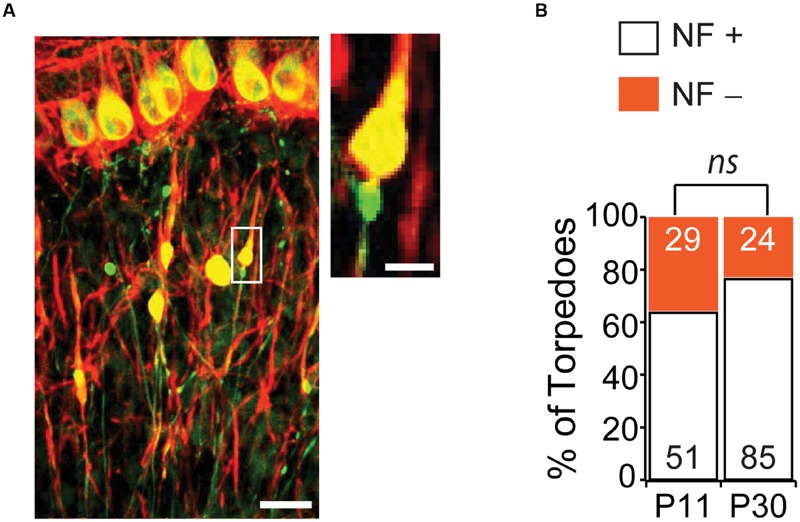
**The majority of Purkinje cell torpedoes contain neurofilament (NF). (A)** Sample image showing Purkinje cells and their axons (green) and neurofilament (red). Co-labeling appears yellow. Most torpedoes are neurofilament (NF) positive, although some are not. Inset shows even neighboring torpedoes on the same axon can be NF+ and NF-. Scale bar in left image, 20 μm; Scale bar in inset, 5 μm. **(B)** Quantification of NF+ (white bars) and NF- (orange bars) at P11 and P30. A sizeable minority of torpedoes are NF-, especially at P11, suggesting that there may be two populations of developmental torpedoes. Significance determined by Mann–Whitney *U* test, *ns P* > 0.05.

One striking feature of disease-related torpedoes that has been observed in several diseases is that they are enriched in disorganized neurofilament (Petito et al., 1973; Yagishita, 1978; Mann et al., 1980; Louis et al., 2009), which may be associated with alterations in axonal transport. We wondered whether developmental torpedoes also contained neurofilament. To address this, we labeled neurofilament 200 kD (NF) and counted the torpedoes that were positive (NF+) and negative (NF-) at both P11 and P30 (**Figure 6A**). At the peak of Purkinje cell torpedo density at P11, although the majority of Purkinje cell axon torpedoes were NF+ (51/80, or 64% of torpedoes), there was a significant fraction that were not (29/80, or 36%; **Figure 6B**). Sometimes neurofilament positive and negative torpedoes were next to each other on the same axon (**Figure 6A**, inset), suggesting that these two types of torpedoes may have distinct functions that are locally determined. Consistent with this, NF+ torpedoes were significantly larger than NF- torpedoes (NF+ length: 7.6 ± 0.3 μm; NF- length: 6.1 ± 0.2 μm; significantly different, *P* = 0.0001; NF+ width: 3.4 ± 0.2 μm; NF- width 2.7 ± 0.1 μm; significantly different, *P* = 0.0007). Later in development at P30, the proportion of NF+ torpedoes had increased slightly although not significantly from P11 (NF+: 85/109, or 78% of torpedoes; NF-: 24/109, or 22% of torpedoes; not significantly different from P11, *P* = 0.09). Similar to what was observed at P11, NF+ torpedoes were also larger than NF- at P30 (NF+ length: 8.9 ± 0.3 μm; NF- length: 7.5 ± 0.6 μm; significantly different, *P* = 0.036; NF+ width: 4.7 ± 0.2 μm; NF- width: 3.5 ± 0.3 μm; significantly different, *P* = 0.003). These data raise the possibility that there may be functional subpopulations of developmental torpedoes, with the majority staining positive for neurofilament, and a significant minority that do not, in contrast to reports of disease-related torpedoes, where neurofilament accumulation in torpedoes is robust (Petito et al., 1973; Yagishita, 1978; Mann et al., 1980; Louis et al., 2009). An alternate explanation is that that the distinction between NF+ and NF- torpedoes is less distinct, with a continuum of neurofilament content in torpedoes ranging from low to high, rather than two separate populations.

**FIGURE 7:**
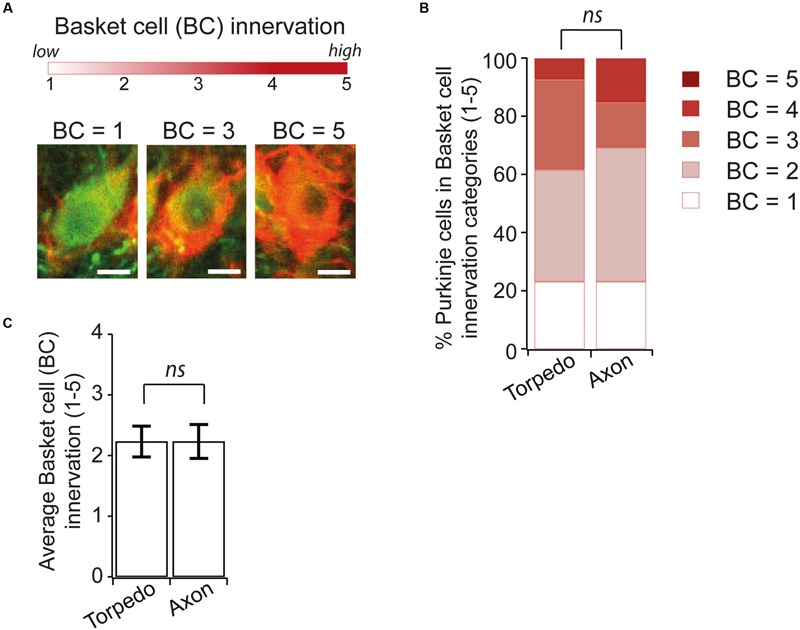
**Similar Basket cell innervation of Purkinje cells with and without developmental torpedoes. (A)** We are analyzing the extent of Basket cell (BC) innervation on individual Purkinje cell somata, where BC = 1 is low, and BC = 5 is high innervation. Sample images showing representative Purkinje cells (green) innervated by Basket cells (labeled red for neurofilament) for low (BC = 1), medium (BC = 3) and high (BC = 5) innervation. Scale bar, 5 μm. **(B)** We traced torpedoes back to their parent Purkinje cell and measured the innervation of BC cells with and without a torpedo on their axon (Torpedo and axon, respectively). % of Purkinje cell with different basket cell innervation was similar in both cases. **(C)** The average Purkinje cell innervation by Basket cells was not significantly different when a torpedo was or was not present (Torpedo: *N* = 13; Axon: *N* = 13; not significantly different, Mann–Whitney *U* test, *P* = 0.92). *ns P* > 0.05.

Recent studies have identified changes in basket cell innervation of Purkinje cells that contain axonal torpedoes in disease states (Erickson-Davis et al., 2010; Kuo et al., 2013). Since basket cell innervation of Purkinje cells is maturing during postnatal development (Ichikawa et al., 2011), we thought that such changes might be associated with developmental torpedoes as well. We identified Purkinje cell neurons that had axonal torpedoes on their axon and examined the extent of basket cell innervation of the parent Purkinje cell [**Figure 7A** shows examples of low (score = 1), medium (score = 3), and high (score = 5) degree of innervation], and compared this with similar neighboring neurons that did not have a torpedo on their proximal axon, using neurofilament labeling to measure basket cell innervation density, since basket cell processes contain neurofilament (Erickson-Davis et al., 2010). In contrast to what has been observed in essential tremor-related torpedoes (Erickson-Davis et al., 2010; Kuo et al., 2013), we found no differences between axonal-torpedo-containing Purkinje cells and those without axonal torpedoes, with both groups being similarly innervated by basket cell processes (**Figures 7B,C**; average basket cell innervation density, scored in arbitrary units from 1 to 5, of Purkinje cells with torpedoes: 2.23 ± 0.26; *N* = 13; average basket cell innervation of Purkinje cells without torpedoes: 2.23 ± 0.28; *N* = 13; not significantly different, *P* = 0.92). Aberrant basket cell innervation of Purkinje cells harboring disease-related axonal torpedoes suggests that circuit rewiring is associated with those neurons. In contrast, our results suggest that developmental torpedoes are not associated with differences in rewiring, but rather appear normally during normal development. Thus, although morphologically similar, there appears to be differences between developmental and disease-related torpedoes.

### Normal Developmental Torpedoes in a Mouse Model of SCA6

**FIGURE 8:**
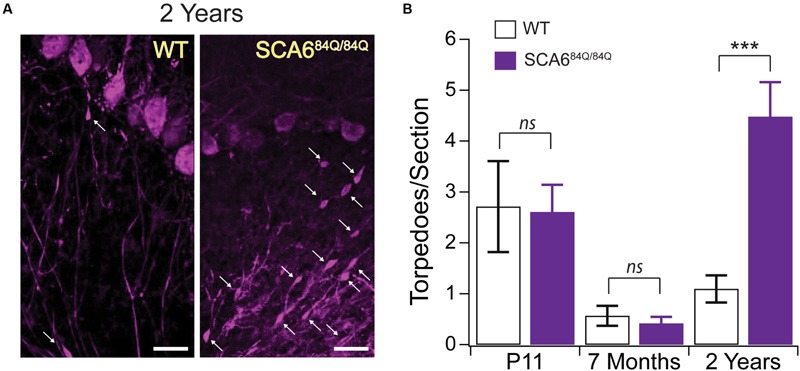
**Developmental torpedoes appear normal in SCA6^84Q/84Q^ mice that later develop disease-related torpedoes. (A)** Sample images of Purkinje cells (purple, labeled with calbindin) from 2-year-old WT (left) and litter-matched SCA6^84Q/84Q^ mice that have more disease-related Purkinje cell axonal torpedoes. White arrows point to torpedoes. Scale bar, 20 μm. **(B)** Although the number of Purkinje cell axonal torpedoes are high in SCA6^84Q/84Q^ mice (purple) compared to WT mice (white bars) at an age when Purkinje cell death is observed (right) (Jayabal et al., 2015), there are no statistically significant differences in the number of torpedoes observed during development at P11 in these mice (left), and torpedo numbers are low in 7-month-old mice when disease symptoms first appear (middle) (Jayabal et al., 2015). These results suggest that developmental torpedoes are not directly related to disease-related torpedoes, as they appear at normal levels in late-onset disease models. Significance determined by Student’s *t*-test, ^∗∗∗^*P* < 0.001, *ns P* > 0.05.

To understand whether a relationship exists between developmental and disease-related torpedoes, we examined Purkinje cell axons at several time points in a mouse model of SCA6, since torpedoes have been seen in postmortem tissue from SCA6 patients (Sasaki et al., 1998; Yang et al., 2000). We used SCA6^84Q/84Q^ mice harboring an expanded polyglutamine (poly-Q) repeat which have been shown to display ataxic symptoms at 7 months old (Watase et al., 2008; Jayabal et al., 2015). We have previously shown that there is no Purkinje cell loss at 7 months when disease symptoms are first observed, although Purkinje cell loss is detectable at 2 years (Jayabal et al., 2015). We first wondered whether disease-related torpedoes would be observed in these mice at 2 years. To address this, we labeled Purkinje cells with calbindin (**Figure 8A**) and counted the number of torpedoes we observed on Purkinje cell axons in both SCA6^84Q/84Q^ and litter-matched wild-type (WT) controls. We found that torpedo number was greatly increased at 2-years in SCA6^84Q/84Q^ compared to WT mice as predicted from human postmortem studies (2 year WT: 1.09 ± 0.27 torpedoes/section; 2 year SCA6^84Q/84Q^: 4.48 ± 0.68 torpedoes/section; significantly different, *P* < 0.0001) (Sasaki et al., 1998; Yang et al., 2000). Interestingly, we have recently shown that transient functional and morphological changes occur during postnatal development in the developing SCA6^84Q/84Q^ cerebellum at the age when we observe developmental torpedoes (Jayabal et al., 2016b). This led us to wonder whether developmental torpedoes might be altered in these mice as well, since this might suggest that they are related to later pathophysiology. We found, however, that developmental torpedo density was normal in P11 SCA6^84Q/84Q^ mice compared to WT, suggesting that they are not directly related to later pathophysiology (P11 WT: 2.71 ± 0.90 torpedoes/section; P11 SCA6^84Q/84Q^: 2.61 ± 0.54 torpedoes/section; not significantly different, *P* = 0.92; **Figure 8B**, left). We also measured the density of Purkinje cell torpedoes in SCA6^84Q/84Q^ and litter-matched mice at 7 months, when cerebellar-related motor deficits are observed without detectable Purkinje cell loss (Jayabal et al., 2015, 2016a), and found that torpedo density was low at 7 months and similar in both WT and SCA6^84Q/84Q^ mice (7 month WT: 0.57 ± 0.20 torpedoes/section; 7 month SCA6^84Q/84Q^: 0.42 ± 0.13 torpedoes/section; not significantly different; *P* = 0.53; **Figure 8B**, middle). Note that we observe higher torpedo numbers in P11 mice than 7-month-old mice in both WT and SCA6^84Q/84Q^ mice, which is consistent with our observations of a transient developmental peak of torpedoes at P11 (**Figure 1**). These data suggest that developmental torpedoes are unlikely to be linked to later disease-related torpedoes and do not by themselves contribute to later pathogenesis. They furthermore suggest that disease-related torpedoes are not an early symptom of SCA6, but rather are observed later during disease progression, at the same time as Purkinje cell loss.

## Discussion

We observed Purkinje cell axonal torpedoes that occurred in the developing mouse cerebellum of both L7-tau-GFP and C57Bl6/J mice, peaking at P11. These torpedoes appear similar to the developmental torpedoes that have been observed in developing rat cerebellum (Gravel et al., 1986). We showed that developmental Purkinje cell axonal torpedoes occur almost exclusively after developmental cell death has occurred when the total density of Purkinje cells was static in Lobule III. Purkinje cell developmental torpedoes were seldom associated with an axon collateral branch point, and microglia were not enriched near developmental torpedoes, suggesting that they are not likely to be associated with axonal pruning. The majority of developmental torpedoes did not label for an inhibitory presynaptic marker, which suggests that they are not the presynaptic element of synapses. Like torpedoes associated with diseases, we found that developmental torpedoes were myelinated and most were enriched with neurofilament, although the presence of a sizeable fraction of non-neurofilament containing torpedoes suggests that there may be two subpopulations of developmental torpedoes. This will be interesting to explore in future studies. Finally, we report that although aged Purkinje cells display disease-related torpedoes in a mouse model of SCA6, we see no differences in the number of developmental torpedoes in these mice, nor are disease-related torpedoes elevated during early disease stages when motor deficits are first observed. Taken together, these results suggest that developmental torpedoes are not pathophysiological in SCA6 mice but rather represent a normal transient morphological feature during the formation of the cerebellar microcircuit.

What function might transient developmental torpedoes serve in the cerebellum? Our findings of neurofilament accumulation in a subset of developmental torpedoes which appear mostly on myelinated axons, suggest that they are involved in normal axonal function, and do not interfere with axonal propagation, for instance, although functional studies are necessary to test this directly. While developmental torpedoes may serve a role in axonal refinement, which is underway at the time point that they are observed (Gianola et al., 2003), the absence of microglia enrichment around developmental torpedoes, and absence of enrichment at axonal collateral branch points argues against this, or at least suggests that their role in axonal refinement is complex. It is thus premature to speculate what role developmental torpedoes play, although our evidence suggests that they are not occurring on Purkinje cells that undergo developmental apoptosis. Further studies will be needed to elucidate their function.

Why do developmental torpedoes appear transiently a few days after birth and then decrease during a few postnatal weeks of development? The age at which torpedoes occur is a time of great restructuring in the developing cerebellum (McKay and Turner, 2005; van Welie et al., 2011; Hashimoto and Kano, 2013; White and Sillitoe, 2013). Several such transient changes have been identified in developing brain circuits that are thought to be involved in its proper development, including transient depolarization by GABAergic innervation (Ben-Ari, 2001, 2002), and transient electrical synapses (Fulton, 1995). Indeed, just in the developing cerebellum, transient presynaptic miniature currents (Trigo et al., 2010), and transient Purkinje – Purkinje synapses that mediate early network activity [(Watt et al., 2009) but see (Witter et al., 2016)] have been shown. Thus, developmental torpedoes appear to represent a normal morphological specialization during the development of Purkinje cell axons. It will be interesting to determine the temporal properties of developmental torpedoes: how long they persist on an axon, whether they are mobile or not, and what triggers their appearance and disappearance on an axon, since this may give us deeper insight into their function.

Superficially, developmental torpedoes appear to resemble disease-related torpedoes, since they have similar morphology, both contain neurofilament, and both occur on myelinated or unmyelinated axons. It is thus possible that similar physiological conditions exist transiently during development and in several diseases cause similar axonal structures in these conditions. However, careful analysis of developmental torpedoes suggests that there are differences between them and disease-related torpedoes that may be functionally meaningful. For instance, while to our knowledge the accumulation of neurofilament in disease-related torpedoes is robust, we see a significant minority of developmental torpedoes that do not appear to contain neurofilament, and these torpedoes were smaller, suggesting that our population of developmental torpedoes is heterogenous and may have distinct functions. However, an alternate explanation would suggest that there exist torpedoes with a range of neurofilament from low to high, rather than two distinct populations, which would suggest that developmental torpedoes may not be distinct from disease-related torpedoes in this manner. In the disease literature, disease-related torpedoes are most commonly observed on non-myelinated axons (Yagishita, 1978; Louis et al., 2009), which is in stark contrast to our developmental torpedoes, where most torpedoes are myelinated, yet another apparent difference between developmental and disease-related torpedoes. Interestingly, disease-related torpedoes are associated with enhanced basket cell inhibitory input onto the parent Purkinje cell (Erickson-Davis et al., 2010; Kuo et al., 2013), which we thought might also occur for developmental torpedoes since basket cell innervation is forming at the ages when developmental torpedoes are observed (Ichikawa et al., 2011). However, we observed no differences in the extent of basket cell innervation between Purkinje cells with torpedoes and without. This suggests that torpedoes may not affect the cerebellar microcircuit to the same extent as disease-related torpedoes. Given the differences existing in the cerebellar microcircuit during development and during disease, it seems more likely that these superficially similar morphological structures have different properties and serve different functions. In a similar vein, although disease-related torpedoes are observed in several diseases, there is evidence that they may have distinct properties in different diseases (Louis et al., 2014). It remains possible, however, that the differences we have identified do not strongly influence cerebellar function and that the similarities between developmental and disease-related torpedoes are enough to produce similar functional consequences. Future experiments are required to resolve this question.

Are developmental torpedoes, which closely resemble disease-related torpedoes, somehow involved in cellular pathophysiology? Our data in normal L7-tau-GFP and C57Bl6/J mice suggest that they are not, but we wanted to address this by examining developmental torpedoes in a disease model that shows enhanced disease-related torpedoes at later ages. We used a mouse model of SCA6 that we have previously characterized in our lab to address this. These mice show initial motor symptoms at 7 months, which progressively worsen (Jayabal et al., 2015). Purkinje cell death is not observed at 7 months, but is seen at 2-years-old (Jayabal et al., 2015). We found that although disease-related torpedoes were highly enriched in 2-year-old mice when Purkinje cell death was observed, there were no differences in torpedo numbers at P11, suggesting that developmental torpedoes are not related to later disease-onset torpedoes. Interestingly, torpedo numbers were elevated at P11 compared to 7 months, consistent with the existence of a transient developmental peak in these mice. Although disease-related torpedoes are likely to differ in different diseases (Louis et al., 2014), but these results suggest that developmental torpedoes are normal and non-pathological, since they appear at normal levels in a mouse that later shows high levels of disease-related torpedoes at old age.

At present, we lack a good understanding of the function of torpedoes, and how they act in Purkinje cells. Interestingly, careful analysis of disease-related torpedoes in essential tremor brains reveals that these structures are simply one of a number of related morphometric changes occurring in Purkinje cells (Babij et al., 2013), suggesting that although perhaps the most notable morphological change, they may not be the most salient change for cerebellar function. What role developmental torpedoes play in circuit formation remains to be seen, but our findings argue that it is not a pathological role. One interpretation of our data suggests that there may be different populations of developmental torpedoes, which might thus serve more than one function in the developing brain. To understand the role of developmental torpedoes, we will need to have a deeper understanding of their functional properties and how they contribute to cerebellar development.

## Author Contributions

LL performed immunocytochemistry, collected and analyzed the data, made figures, and helped to write the manuscript, DL-O performed immunocytochemistry, collected and analyzed the data, and helped to write the manuscript, AY performed immunocytochemistry and analyzed the data, SJ prepared tissue, collected and analyzed the data, and helped to write the manuscript, SQ performed immunocytochemistry and analyzed the data, and AW analyzed the data, made figures, and wrote the manuscript.

## Conflict of Interest Statement

The authors declare that the research was conducted in the absence of any commercial or financial relationships that could be construed as a potential conflict of interest.

## References

[B1] Babij R. Lee M. Cortes E. Vonsattel J. P. Faust P. L. Louis E. D. (2013). Purkinje cell axonal anatomy: quantifying morphometric changes in essential tremor versus control brains. *Brain* 136 3051–3061. 10.1093/brain/awt238 24030953 PMC3784286

[B2] Baurle J. Grusser-Cornehls U. (1994). Axonal torpedoes in cerebellar Purkinje cells of two normal mouse strains during aging. *Acta Neuropathol.* 88 237–245. 10.1007/BF00293399 7810294

[B3] Ben-Ari Y. (2001). Developing networks play a similar melody. *Trends Neurosci.* 24 353–360. 10.1016/S0166-2236(00)01813-0 11356508

[B4] Ben-Ari Y. (2002). Excitatory actions of gaba during development: the nature of the nurture. *Nat. Rev. Neurosci.* 3 728–739. 10.1038/nrn920 12209121

[B5] Bouman L. (1918). *Die Histopathologie Der Psychosen*. Amsterdam: Springer.

[B6] Chamberland S. Evstratova A. Toth K. (2014). Interplay between synchronization of multivesicular release and recruitment of additional release sites support short-term facilitation at hippocampal mossy fiber to CA3 pyramidal cells synapses. *J. Neurosci.* 34 11032–11047. 10.1523/JNEUROSCI.0847-14.2014 25122902 PMC6705252

[B7] di Penta A. Moreno B. Reix S. Fernandez-Diez B. Villanueva M. Errea O. (2013). Oxidative stress and proinflammatory cytokines contribute to demyelination and axonal damage in a cerebellar culture model of neuroinflammation. *PLoS ONE* 8:e54722 10.1371/journal.pone.0054722 PMC3576396 23431360

[B8] Duan Y. Dong S. Gu F. Hu Y. Zhao Z. (2012). Advances in the pathogenesis of Alzheimer’s disease: focusing on tau-mediated neurodegeneration. *Transl. Neurodegener.* 1:24 10.1186/2047-9158-1-24 PMC3598890 23241453

[B9] Erickson-Davis C. R. Faust P. L. Vonsattel J. P. Gupta S. Honig L. S. Louis E. D. (2010). “Hairy baskets” associated with degenerative Purkinje cell changes in essential tremor. *J. Neuropathol. Exp. Neurol.* 69 262–271. 10.1097/NEN.0b013e3181d1ad04 20142764 PMC2865233

[B10] Fulton B. P. (1995). Gap junctions in the developing nervous system. *Perspect. Dev. Neurobiol.* 2 327–334. 7538866

[B11] Gianola S. Savio T. Schwab M. E. Rossi F. (2003). Cell-autonomous mechanisms and myelin-associated factors contribute to the development of Purkinje axon intracortical plexus in the rat cerebellum. *J. Neurosci.* 23 4613–4624. 12805301 10.1523/JNEUROSCI.23-11-04613.2003 PMC6740793

[B12] Gravel C. Leclerc N. Plioplys A. Hawkes R. B. (1986). Focal axonal swellings in rat cerebellar Purkinje cells during normal development. *Brain Res.* 363 325–332. 10.1016/0006-8993(86)91018-8 3510690

[B13] Hashimoto K. Kano M. (2013). Synapse elimination in the developing cerebellum. *Cell Mol. Life Sci* 70 4667–4680. 10.1007/s00018-013-1405-2 23811844 PMC3830199

[B14] Hirano A. Dembitzer H. M. Ghatak N. R. Fan K. J. Zimmerman H. M. (1973). On the relationship between human and experimental granule cell type cerebellar degeneration. *J. Neuropathol. Exp. Neurol.* 32 493–502. 10.1097/00005072-197310000-00002 4128006

[B15] Ichikawa R. Yamasaki M. Miyazaki T. Konno K. Hashimoto K. Tatsumi H. (2011). Developmental switching of perisomatic innervation from climbing fibers to basket cell fibers in cerebellar Purkinje cells. *J. Neurosci.* 31 16916–16927. 10.1523/JNEUROSCI.2396-11.2011 22114262 PMC6623856

[B16] Jankowski J. Miething A. Schilling K. Baader S. L. (2009). Physiological purkinje cell death is spatiotemporally organized in the developing mouse cerebellum. *Cerebellum* 8 277–290. 10.1007/s12311-009-0093-9 19238501

[B17] Jayabal S. Chang H. H. V. Cullen K. E. Watt A. J. (2016a). 4-Aminopyridine alleviates ataxia and reverses cerebellar output deficiency in a mouse model of spinocerebellar ataxia type 6. *Sci. Rep.* 6 29489 10.1038/srep29489 PMC4933933 27381005

[B18] Jayabal S. Ljungberg L. Erwes T. Cormier A. Quilez S. El Jaouhari S. (2015). Rapid onset of motor deficits in a mouse model of spinocerebellar ataxia type 6 precedes late cerebellar degeneration. *eNeuro* 2 1–18. 10.1523/ENEURO.0094-15.2015 PMC4697081 26730403

[B19] Jayabal S. Ljungberg L. Watt A. J. (2016b). Transient cerebellar alterations during development prior to obvious motor phenotype in a mouse model of spinocerebellar ataxia type 6. *J. physiol.* 10.1113/JP273184 [Epub ahead of print]. PMC5285638 27531396

[B20] Kato G. Inada H. Wake H. Akiyoshi R. Miyamoto A. Eto K. (2016). Microglial contact prevents excess depolarization and rescues neurons from excitotoxicity. *eNeuro* 3 1–9. 10.1523/ENEURO.0004-16.2016 PMC4916329 27390772

[B21] Kato T. Hirano A. (1985). A Golgi study of the proximal portion of the human Purkinje cell axon. *Acta Neuropathol.* 68 191–195. 10.1007/BF00688636 3909727

[B22] Krstic D. Knuesel I. (2013). Deciphering the mechanism underlying late-onset Alzheimer disease. *Nat. Rev. Neurol.* 9 25–34. 10.1038/nrneurol.2012.236 23183882

[B23] Kuo S. H. Tang G. Louis E. D. Ma K. Babji R. Balatbat M. (2013). Lingo-1 expression is increased in essential tremor cerebellum and is present in the basket cell pinceau. *Acta Neuropathol.* 125 879–889. 10.1007/s00401-013-1108-7 23543187 PMC3663903

[B24] Lariviere R. Gaudet R. Gentil B. J. Girard M. Conte T. C. Minotti S. (2015). Sacs knockout mice present pathophysiological defects underlying autosomal recessive spastic ataxia of Charlevoix-Saguenay. *Hum. Mol. Genet.* 24 727–739. 10.1093/hmg/ddu491 25260547 PMC4291249

[B25] Light K. E. Belcher S. M. Pierce D. R. (2002). Time course and manner of Purkinje neuron death following a single ethanol exposure on postnatal day 4 in the developing rat. *Neuroscience* 114 327–337. 10.1016/S0306-4522(02)00344-5 12204202

[B26] Louis E. D. Faust P. L. Ma K. J. Yu M. Cortes E. Vonsattel J. P. (2011). Torpedoes in the cerebellar vermis in essential tremor cases vs. controls. *Cerebellum* 10 812–819. 10.1007/s12311-011-0291-0 21656041

[B27] Louis E. D. Kuo S. H. Vonsattel J. P. Faust P. L. (2014). Torpedo formation and Purkinje cell loss: modeling their relationship in cerebellar disease. *Cerebellum* 13 433–439. 10.1007/s12311-014-0556-5 24590661 PMC4077970

[B28] Louis E. D. Vonsattel J. P. Honig L. S. Ross G. W. Lyons K. E. Pahwa R. (2006). Neuropathologic findings in essential tremor. *Neurology* 66 1756–1759. 10.1212/01.wnl.0000218162.80315.b9 16769958

[B29] Louis E. D. Yi H. Erickson-Davis C. Vonsattel J. P. Faust P. L. (2009). Structural study of Purkinje cell axonal torpedoes in essential tremor. *Neurosci. Lett.* 450 287–291. 10.1016/j.neulet.2008.11.043 19047012 PMC2662443

[B30] Mann D. M. Stamp J. E. Yates P. O. Bannister C. M. (1980). The fine structure of the axonal torpedo in Purkinje cells of the human cerebellum. *Neurol. Res.* 1 369–378. 10.1080/01616412.1980.11739567 6107881

[B31] McKay B. E. Turner R. W. (2005). Physiological and morphological development of the rat cerebellar Purkinje cell. *J. Physiol.* 567 829–850. 10.1113/jphysiol.2005.089383 16002452 PMC1474219

[B32] Perez-Pouchoulen M. VanRyzin J. W. McCarthy M. M. (2015). Morphological and phagocytic profile of microglia in the developing rat cerebellum(1,2,3). *eNeuro* 2 10.1523/ENEURO.0036-15.2015 PMC4596010 26464992

[B33] Petito C. K. Hart M. N. Porro R. S. Earle K. M. (1973). Ultrastructural studies of olivopontocerebellar atrophy. *J. Neuropathol. Exp. Neurol.* 32 503–522. 10.1097/00005072-197310000-00003 4357157

[B34] Pioro E. P. Cuello A. C. (1988). Purkinje cells of adult rat cerebellum express nerve growth factor receptor immunoreactivity: light microscopic observations. *Brain Res.* 455 182–186. 10.1016/0006-8993(88)90131-X 2843259

[B35] Pologruto T. A. Sabatini B. L. Svoboda K. (2003). ScanImage: flexible software for operating laser scanning microscopes. *Biomed. Eng. Online* 2 13 10.1186/1475-925X-2-13 PMC161784 12801419

[B36] Pont-Lezica L. Bechade C. Belarif-Cantaut Y. Pascual O. Bessis A. (2011). Physiological roles of microglia during development. *J. Neurochem.* 119 901–908. 10.1111/j.1471-4159.2011.07504.x 21951310

[B37] Ramon Y. Cajal S. (1991). *Cajal’s Degeneration and Regeneration of the Nervous System*. New York, NY: Oxford University Press.

[B38] Rossi F. Borsello T. Strata P. (1994). Exposure to kainic acid mimics the effects of axotomy in cerebellar Purkinje cells of the adult rat. *Eur. J. Neurosci.* 6 392–402. 10.1111/j.1460-9568.1994.tb00282.x 8019676

[B39] Sarna J. R. Hawkes R. (2011). Patterned Purkinje cell loss in the ataxic sticky mouse. *Eur. J. Neurosci.* 34 79–86. 10.1111/j.1460-9568.2011.07725.x 21645134

[B40] Sasaki H. Kojima H. Yabe I. Tashiro K. Hamada T. Sawa H. (1998). Neuropathological and molecular studies of spinocerebellar ataxia type 6 (SCA6). *Acta Neuropathol.* 95 199–204. 10.1007/s004010050787 9498057

[B41] Sekirnjak C. Vissel B. Bollinger J. Faulstich M. du Lac S. (2003). Purkinje cell synapses target physiologically unique brainstem neurons. *J. Neurosci.* 23 6392–6398. 12867525 10.1523/JNEUROSCI.23-15-06392.2003 PMC6740533

[B42] Sotelo C. (1990). Axonal abnormalities in cerebellar Purkinje cells of the ‘hyperspiny Purkinje cell’ mutant mouse. *J. Neurocytol.* 19 737–755. 10.1007/BF01188042 2077114

[B43] Takahashi N. Iwatsubo T. Nakano I. Machinami R. (1992). Focal appearance of cerebellar torpedoes associated with discrete lesions in the cerebellar white matter. *Acta Neuropathol.* 84 153–156. 10.1007/BF00311388 1523970

[B44] Takeuchi I. K. Aoki E. Takeuchi Y. K. (1995). Axonal swellings in cerebellar white matter of groggy mutant rat. *Acta Neuropathol.* 90 486–492. 10.1007/BF00294810 8560982

[B45] Trigo F. F. Bouhours B. Rostaing P. Papageorgiou G. Corrie J. E. Triller A. (2010). Presynaptic miniature GABAergic currents in developing interneurons. *Neuron* 66 235–247. 10.1016/j.neuron.2010.03.030 20435000

[B46] van Welie I. Smith I. T. Watt A. J. (2011). The metamorphosis of the developing cerebellar microcircuit. *Curr. Opin. Neurobiol.* 21 245–253. 10.1016/j.conb.2011.01.009 21353528 PMC3096781

[B47] Volk B. Kirchgassner N. (1985). Damage of Purkinje cell axons following chronic phenytoin administration: an animal model of distal axonopathy. *Acta Neuropathol.* 67 67–74. 10.1007/BF00688125 4024872

[B48] Watase K. Barrett C. F. Miyazaki T. Ishiguro T. Ishikawa K. Hu Y. (2008). Spinocerebellar ataxia type 6 knockin mice develop a progressive neuronal dysfunction with age-dependent accumulation of mutant CaV2.1 *channels*. *Proc. Natl. Acad. Sci. U.S.A.* 105 11987–11992. 10.1073/pnas.0804350105 18687887 PMC2503926

[B49] Watt A. J. Cuntz H. Mori M. Nusser Z. Sjöström P. J. Häusser M. (2009). Traveling waves in developing cerebellar cortex mediated by asymmetrical Purkinje cell connectivity. *Nat. Neurosci.* 12 463–473. 10.1038/nn.2285 19287389 PMC2912499

[B50] White J. J. Sillitoe R. V. (2013). Development of the cerebellum: from gene expression patterns to circuit maps. *Wiley Interdiscip. Rev. Dev. Biol.* 2 149–164. 10.1002/wdev.65 23799634

[B51] Witter L. Rudolph S. Pressler R. T. Lahlaf S. I. Regehr W. G. (2016). Purkinje cell collaterals enable output signals from the cerebellar cortex to feed back to Purkinje cells and interneurons. *Neuron* 91 312–319. 10.1016/j.neuron.2016.05.037 27346533 PMC4969194

[B52] Yagishita S. (1978). Morphological investigations on axonal swellings and spheroids in various human diseases. *Virchows Arch. A Pathol. Anat. Histol.* 378 181–197. 10.1007/BF00427359 150108

[B53] Yang Q. Hashizume Y. Yoshida M. Wang Y. Goto Y. Mitsuma N. (2000). Morphological Purkinje cell changes in spinocerebellar ataxia type 6. *Acta Neuropathol.* 100 371–376. 10.1007/s004010000201 10985694

